# Suicide Investigations in Adult Community Mental Health Services: Mitigation of the Fear of Blame as a Barrier to Organisational Learning

**DOI:** 10.1111/inm.70136

**Published:** 2025-09-04

**Authors:** Helen Haylor, Tony Sparkes, Gerry Armitage, Keith Double, Lisa Edwards

**Affiliations:** ^1^ First Response Bradford District Care NHS Foundation Trust Bradford UK; ^2^ Faculty of Management, Law and Social Sciences University of Bradford Bradford UK; ^3^ Research and Development Department Bradford District Care NHS Foundation Trust Bradford UK; ^4^ Faculty of Health Studies University of Bradford Bradford UK; ^5^ Patient and Carer Experience and Involvement Team Bradford District Care NHS Foundation Trust Bradford UK

**Keywords:** mental health services, organisational learning, suicide investigations

## Abstract

Effective suicide prevention strategies in community mental health services demand high standards of patient safety. The nature of suicide is complex and uncertain. However, learning needs to be sensitive to the fear of blame. Little is known about how health services investigate suicides post hoc, how they examine why a suicide might have occurred, and how they generate any organisational learning that might improve patient safety. The aim of this novel qualitative study was to explore the accounts of key people involved in the serious incident investigation process regarding the subsequent organisational learning. Carers, clinicians, investigators and senior managers were recruited via regional and national networks; data were collected through focus groups and individual interviews. The dominant themes that emerged from this analysis did not reflect the tenor of the literature related to the investigative process and organisational learning, accepting that the literature is relatively sparse. A reflexive thematic analysis developed an understanding of mitigations against a fear of blame which appeared protective of all participants' positions, including those specific to suicide risk. We argue that mitigation operated as a barrier to organisational learning and improving patient safety in adult community mental health services. The findings are discussed in the context of organisational culture, learning and wider system thinking. The concept of mitigation against a fear of blame constructs new insights into this ambiguous and emotionally demanding sphere of patient safety.

## 1 Introduction

In England, the Patient Safety Incident Response Framework (PSIRF; NHS England 2022) was introduced to replace the Serious Incident Framework (NHS England 2015) as a way of improving patient safety standards. However, it is a generic tool and not tailored to the complexities of mental health settings. Recently published reviews raised concerns about patient safety in community mental health services: Averill et al. (2023) reported a paucity of applied patient safety research; Fröding et al. (2022) argued for a ‘paradigm shift’ in how organisations learn from suicides, and Haylor et al. (2024) reported a lack of evidence around how organisations investigate and learn from suicides in adult community mental health services (ACMHS). The latter also highlighted a particular complexity surrounding deaths from suicide, the limited evidence base underpinning the dominant investigative method—typically Root Cause Analysis (RCA), and long‐standing concerns about its appropriateness (Vrklevski et al. 2018). Much of this concern centres around the unpredictability of suicide (Franklin et al. 2017) and it being unsuitable for the linear causal mechanisms which RCA assumes. Fundamentally, the reasons an individual decides to take their own life can never be entirely knowable. However, as indicated in Kapur et al.'s (2025) recent commentary, the work of Cero et al. (2021) is relevant here, acknowledging that while causal inferences cannot be confidently made, evidence‐informed appraisals of possible contributory factors are of paramount importance in driving up the standards of patient safety.

Parallel to the emergent focus on patient safety in mental health services, clinical approaches to suicide risk assessment (RA) have been subject to considerable scrutiny and have led to a substantial shift from predictive scales and checklists to individualised, formulation‐based preventative approaches (Hawton et al. 2022; Pisani et al. 2016). Furthermore, a quasi‐empirical study explored the quality of suicide RAs prior to suicide, demonstrating poor quality in cases where patient risk was labelled ‘low’ (Rahman et al. 2013); although we are not aware of any confirmatory research undertaken by these authors. Furthermore, Haylor et al. (2024) reported a lack of critical appraisal around RA in investigative processes following ACMHS suicides, undermining the quality of organisational learning.

Cohen (2013) draws upon Menzies‐Lyth (1989) to argue that serious incident investigations (SIIs) can inadvertently function as a defensive social system for managing organisational and clinician anxieties. Approaches taken to suicide RA have also been discussed in relation to anxiety management (Hawton et al. 2022). As a potential solution, the need for a Restorative Just Culture (RJC) has arisen, underpinned by an aspirational Zero Suicide Framework (ZSF) which would drive the objectives of SIIs (Turner et al. 2020). In common with the UK's PSIRF (NHS England 2022), Turner et al. (2020) also embrace a systems approach to suicide prevention; advocating what is known as Safety 2 (Braithwaite et al. 2015). Both approaches seek to address cultures of blame within patient safety frameworks.

A RJC recognises the potential harms impacting all those close to patients who die by suicide, while simultaneously prioritising organisational learning. Concerns have been expressed around the potential for ZSF's to have a negative effect upon clinician welfare and the potential for further self‐blame post suicide (Mokkenstorm et al. 2018). However, early findings from their implementation process, which held the term ‘zero’ as an aspiration and not a target, indicate some positive impacts on organisational learning and staff experience (Turner et al. 2021). A summary explanation of ZSF and RJC is included in Box 1 and 2 below.

BOX 1 **Summary of the seven elements of the Zero Suicide Framework (adapted from Zero Suicide International 2018).**

**Table:** 

**Lead**: System change occurs with sustained and committed leaders who learn and improve practices following adverse events
**Train**: Train all staff—clinical and non‐clinical—to identify individuals at risk and respond effectively, commensurate with their roles
**Identify**: Screen and assess every new and existing patient for suicidal thoughts and behaviours in an ongoing and systematic way using standardized tools
**Engage**: Patients at risk for suicide agree to actively engage in a package of evidence‐based practices that directly targets their suicidal thoughts and behaviours
**Treat**: Utilize evidence‐based treatments that focus explicitly on reducing suicide risk to keep patients safe and help them thrive
**Transition**: Put policies into action that ensure safe hand‐offs between caregivers and upon discharge
**Improve**: Apply data‐driven quality improvement. Use data to inform system changes that will lead to improved patient outcomes and better care for those at risk

BOX 2 **Summary of response to incidents using a Restorative Just Culture (RJC) framework (adapted from Turner et al. 2020).**

**Table:** 

**Who is hurt?** Consumer, family, carer, clinician and organisation
**What do they need?** Tailored to each individual, consideration may encompass: Support, healing, information, engagement in review and learning
**Obligations and actions** For all affected there are obligations and actions required by the organisation to provide transparency about what has happened, inclusive involvement in review processes, necessary high‐quality learning is made and appropriate emotional support is provided throughout

The empirical research underpinning SIIs is both thin and disparate (Haylor et al. 2024), particularly in relation to those close to the process, and who rely on its rigour and integrity such as mental health clinicians, senior managers and serious incident investigators, and of course carers. In this paper, we report novel themes from a qualitative, exploratory study which set out to capture the views of those closely connected with SIIs, sharing their experiences and views on the potential for organisational learning. The analysis yielded particular themes which were developed around an implicit fear of blame which we argue could have significant implications for the process and outcomes of SIIs.

## 2 Methods

### 2.1 Design

The research design followed a qualitative approach and flowed from our related integrative literature review and narrative synthesis (Haylor et al. 2024). Two carers (KD and LE) were involved from research conceptualisation to final report as part of our multi‐disciplinary research team (HH, TS and GA). We agreed on a multi‐method, sequential study employing focus groups and semi‐structured interviews.

HH, TS, GA and LE had prior experience of conducting qualitative research. Qualitative approaches are positively indicated in the patient safety literature (Albutt et al. 2021; Berzins et al. 2020) and are well‐suited to understanding individual experience. To reduce the subjective biases associated with our own perspectives, we instigated periodic team meetings where individual assumptions, particularly around data analysis, were challenged. Virtual methods were chosen to manage uncertainties in the latter stages of the COVID‐19 pandemic, mindful of the impact of moving from in‐person to virtual methods (Dos Santos Marques et al. 2021; Hensen et al. 2021).

Separate carer and clinician focus groups were planned; the data analysis from these groups would then partly inform subsequent semi‐structured 1:1 interviews with managers and investigators. Focus groups reflected the methodological imperative for people with similar experiences to meet, share and reflect upon their experiences (Kruger and Casey 2014). As one study objective was to best understand the implications of SIIs for organisational learning, we chose the 1:1 interview as a more appropriate tool to develop in‐depth dialogue with investigators and managers who held potentially diverse expectations and specific responsibilities for organisational learning (DeJonckheere and Vaughn 2019). Both methods were judged to be suitable for challenging research topics such as those concerning healthcare systems and iatrogenic harm (Silverio et al. 2022).

### 2.2 Sample

A purposive sampling strategy was used to capture information‐rich cases (Patton 2014), utilising four different groups (clinicians, carers, managers and investigators) who were all contributors, or potential contributors to the SII process and had experience of ACMH SIIs in the last 2 years. We initially sought to recruit from the host Trust.

#### 2.2.1 Clinician Access and Recruitment

Local investigation team leaders identified potential participants. Those interested in taking part were provided with a participant information sheet (PIS) and asked to contact HH for further information. We recruited from both genders (*n* = 4; male = 1/female = 3) and three professional groups: Clinical Psychology (*n* = 1), Social Work (*n* = 1) and Mental Health Nursing (*n* = 2) from the host Trust.

#### 2.2.2 Carer Access and Recruitment

Carers were initially sought from the host Trust. Carers' names and postal addresses were provided by the local investigation team leaders. Carers were sent PISs, with a request to contact HH if interested. This method yielded low numbers (*n* = 2). To enhance participation, we recruited from across England, which encompassed local and national support groups and services; an advertisement was placed in a national charity newsletter, and local bereavement workers were asked to advertise the study. Carers were advised to email HH if interested and wanted a PIS. Four additional carers were recruited via this method, making a total of 6 (male = 1; female = 5).

#### 2.2.3 Manager Access and Recruitment

Relevant managers had been identified by senior leaders of the host Trust. Managers were contacted by HH and provided with a PIS. We recruited 6 managers (male = 2/female = 4).

#### 2.2.4 Investigator Access and Recruitment

Two research team members initially arranged to meet with a group of host Trust investigators, but recruitment was not successful. The sampling frame was adjusted to include two neighbouring Trusts. Potential investigators were approached via their managers and asked to contact HH for a PIS. Three investigators took part in the study (male = 1; female = 2) from two NHS Trusts.

Ethical approval was obtained via HRA and Health and Care Research Wales (HCRW) (IRAS project ID: 298451). All enhancements to the original approach were notified and agreed with HCRW. HH individually met all participants online to gain informed consent.

### 2.3 Data Collection

All focus groups and interviews in the study included questions influenced by our literature review (Haylor et al. 2024). The original design proposed focus groups with carers and clinicians that would further inform the questions taken to individual interviews with managers and investigators. Themes which were developed from the clinicians' focus groups confirmed the questions we had previously identified as important to explore. However, data from the carers' focus group only had a weak pull on further development of the original questions. Nevertheless, the questions originally identified were still deemed relevant.

Due to recruitment difficulties with investigators described above, we decided to amend our study design by speaking with investigators in a focus group instead of individual interviews. We proposed that a focus group setting would assuage any anxieties that may have been present, and this also allowed us to manage the time delays already experienced. HH and TS conducted all focus groups. Carer advisor (KD) was present in the carer focus group to ensure a greater level of empathy with carers and to support researcher sensitivity but did not act in a co‐facilitator role. Focus groups were 60–90 min, facilitated to ensure the voice of all participants, and implemented remotely via Microsoft Teams (MT) and recorded to aid transcription. Focus groups and 1:1 interviews were undertaken in a conversational style supported by various prompt questions. The interviews were undertaken by HH (*n* = 3) and TS (*n* = 3), and were also 60–90 min, and audio recorded via MT. Data was collected between December 2021 and December 2022. Topic guides for each of our participant groups can be found in our Supporting Information S1–S5.

### 2.4 Data Analysis

Audio recordings were transcribed verbatim. Identifying information was removed at transcription and recordings were then destroyed. Transcriptions were checked for accuracy by HH and TS. We undertook a reflexive thematic analysis and followed a systematic, iterative process (Braun and Clarke 2006, 2019).

HH and TS applied and developed codes and themes through an iterative reading of the transcripts (see Table 1). Readings were simultaneously inductive and deductive, reflexively acknowledging the authors' values, skills, experience and training (Braun and Clarke 2021, 39), adding interpretive value and authenticity to the analysis. Our empirical work here was underpinned by our undertaking of an integrative literature review (Haylor et al. 2024), the review increased our sensitivity to the topics which our participants may have found difficult to talk about within the SII process. Following Braun and Clarke's (2006, 2019) guidance, analysis focused on making sense of and interpreting data relative to the research aims, identifying semantic and latent themes. We deemed that developing latent themes was particularly important given the sensitivity of the topic (Silverio et al. 2022). Data triangulation (Carter et al. 2014) from focus groups and 1:1 interviews allowed the development of a more meaningful and comprehensive understanding of participants experience of SIIs.

**TABLE 1:** Phase and description of data analysis (after Braun and Clarke 2006).

	Phase	Description
1	Familiarisation	Iterative reading/re‐reading and provisional ideas
2	Codes	Identifying patterns in the dataset, and systematically demarking these
3	Theme generation	Organising codes into potential themes and allocating data to each theme
4	Theme review/revision	Revising the fit between code and theme; and mapping this across the wider data set
5	Theme definition and naming	Refining and polishing of phase 4, and allocating names to themes
6	Examples	Selecting exemplars of each theme and building these into a meaningful narrative

Our interpretative stance was informed by a constructionist epistemology, noting the everyday use of language in socially constructing (Burr 2015) and making sense of the SII process. Any areas of disagreement were adjudicated by GA. Findings are reported according to COREQ (Tong et al. 2007). See Supporting Information S1.

## 3 Findings

The emotional context of suicide is unavoidable. The traumatic nature of death by suicide was something all participant groups mentioned, as was perhaps unsurprisingly, a fear of blame which was seen as something that needed to be managed. In making sense of these findings, we were therefore mindful of the individual but also the collective experiences within each of the participant groups and the potential impact this may have had to speak openly about this subject within the SII process.

Individual participant groups' overall talk reflected a need to offer some mitigation against the possibility that they had somehow contributed to the suicide or gaps in learning processes (investigators and managers). We understand this as perhaps indicating a need to protect their own well‐being in relation to the death and the SII processes; and specific to the health professionals—their own roles and accountabilities. These mitigations may have been conscious or unconscious.

We developed a theme which relates to a fear of blame in relation to the SII and the death itself. A second theme relates to ‘mitigation’, which we understand as in use by each participant group as a protective strategy.

We present our findings according to each participant group, beginning by sharing those relating to a fear of blame. Initially, this draws from what the participant group themselves reported. To strengthen the credibility of this theme, we include relevant accounts from other participant groups. We then share findings relating to mitigation. Our thematic map is shown in Table 2.

**TABLE 2:** Thematic map.

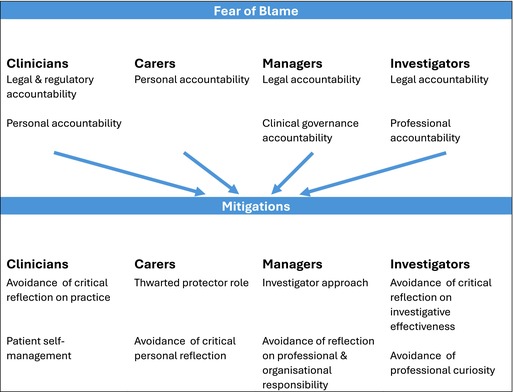

### 3.1 Clinicians

Due to their roles and accountabilities, it is perhaps unsurprising that clinicians' openly acknowledged vulnerability to blame. Clinician vulnerability was also acknowledged by managers.

#### 3.1.1 Legal and Regulatory Accountability

All clinicians spoke of their awareness of the potential for blame as is captured in the following:

You are anxious. Even though I am confident that I'd done everything as I should do, you worry that you can be in for some criticism. And you just want a chance to defend that. Clinician:3

All managers acknowledged that clinicians were likely to fear blame, and this reduced the likelihood of them being open within SSIs:

[It's been] recognised for years that if people feel they're gonna be blamed, they won't say anything. Manager:5

Managers spoke of their commitment to avoid apportioning blame wherever it was appropriate. However, managers also acknowledged the oversight of external regulators in a SII and inherent risk of career‐ending consequences. This left one manager speaking hesitantly about absolute reassurances to a ‘no blame’ approach:

I can count on the fingers of one hand how many times I've seen somebody struck off on the back of an SI, it just doesn't happen very often. Manager:2

#### 3.1.2 Personal Accountability

There were also indications that clinician anxieties extended beyond possible legal implications, but more broadly to personal self‐concept. The following clinician reflected on providing de‐briefs to staff involved in SIIs:

I'm involved in debriefs of people who have been going through that process, and it can really kind of get people to start thinking ‘is that my profession?’ ‘Am I good enough for this?’ ‘Should I do this?’ ‘Am I having the right qualifications?’ So, I think the impact of investigations on staff is something that's really needs to be thought about. I think it's hard to know that somebody that you worked with, that you had a therapeutic relationship, a professional relationship, but you are still [a] human‐being. Clinician:2

It is our understanding that clinicians attempted to manage their doubts and fears when involved in an investigation by using mitigating language regarding their roles and responsibilities.

#### 3.1.3 Avoidance of Critical Reflection on Practice

Clinicians employed more extreme language, constructing a sense of objectivity, making the case that their actions were correct and perhaps defending against any counterargument. All four clinicians spoke about ostensibly valid patient and service considerations which they proposed had influenced their actions. Importantly, they did not identify anything that could have been done differently.

Clinician 1 spoke of how they had been asked by an investigator about their rationale for discharging a patient from ACMHS. The way the clinician described the investigator's approach suggested an increasing defensiveness from the clinician:

When my work was being looked at it was: ‘So you still discharged them?’ ‘Yeah.’ ‘You still discharged and (…) they'd rung XX [crisis service] and they'd been assessed by the Intensive Home Treatment Team?’ And I said ‘That's progress, that's what we want. This is going to be their future care and treatment (…) they don't need anything ongoing. They've got to learn to manage in a crisis, and one of those things is asking for help and that's what they've done.’ Clinician:1

#### 3.1.4 Responsibility for Self‐Management

Two clinicians were articulate when explaining their decision‐making and attributed the deaths to service‐user responsibility. This is captured in Clinician 1's excerpt above and in the following excerpt from Clinician 3. Responsibility is placed upon the capacious patient despite acknowledging ‘high‐risk’ indicators. The clinician had already explained they had referred the patient to the crisis team:

I'd offered this young man quite a range of options (…) all of which he declined (…) this young man ticked a lot of boxes in terms of completing suicide (…) he was the right age, unemployed, substance misuse, the list went on. But none of those were due to acute mental illness. They were very much (…) social issues and poor coping. He turned them down and ultimately (…) he had capacity to make those decisions. Clinician:3

### 3.2 Carers

Interpersonal relationships with carers are important contextual sources of information in making sense of the death, but exploring this would understandably be sensitive. There was little accounting for self‐blame in carers talk, but reference to grief processes was apparent in all participants' accounts.

#### 3.2.1 Personal Accountability

Carers did not talk about fear of blame. However, this excerpt captures a sense of the potential:

To me that feels like a reason sometimes why people are pushed off Section [The Mental Health Act 1983] because the risk then goes (…) back on the family rather than on the Trust. Carer:3

The emotional impact on carers was evident in their behaviour in the focus group, although they made little explicit mention of this. All clinicians, managers and investigators conveyed their awareness and sensitivity to the inevitable grief carers experience. For example:

The more you keep them up to date and spend time with them the better (…) making sure that they're looking after themselves, that they've got information about bereavement services. Investigator:3

And then you're also thinking all the time ‘What about the family? How is this impacting them? How do they feel?’ Clinician:2

One manager went further by stating that carers will be dealing with guilt:

There's going to be families feeling incredibly guilty, feeling incredibly angry, in all of those stages of grief. Manager:2

Our understanding of carers' talk, worked to mitigate against the impact of suicide bereavement including guilt, shame and self‐blame.

#### 3.2.2 Thwarted Protector Role

Carers' talk reflected their capability to save their loved one's life, but mental health services had prevented them. While articulating valid learning points regarding the appropriate involvement of carers in mental healthcare, they also spoke with certainty about the deceased's vulnerability. In the following, carers' talk seemed to function to claim they knew what their loved ones were going to do, and that they had shared this with clinicians:

I knew for weeks. I had the vision of what he was going to do, my heart told me what he was going to do. And he did exactly what I told the doctors and nurses he would do. Carer:1

#### 3.2.3 Avoidance of Personal Reflection

Within carers' talk of the value of what was considered within the SII, there was no reference to how their inter‐personal relationship with their loved one was important in making sense of the death. To support our interpretation of this function, the following excerpt is the only instance where familial experiences are referred to and the carer describes reacting defensively:

They said (…) ‘we don't involve families [in care provision] usually because family is where the issues of these persons usually stem from’. And I was sort of in shock, because I thought, ‘my son's life wasn't perfect by any means, but he had quite a nice childhood’. Carer:4

### 3.3 Managers

Managers clearly hold high levels of accountability regarding suicide, to which they gave serious consideration, although they did not share explicit fears of blame. Carers and clinicians overtly state the potential for blame for managers in seeking to protect organisational reputation.

#### 3.3.1 Legal and Reputational Accountability

Manager's accounts conveyed their awareness of senior levels of oversight of SIIs, citing the quality regulator, commissioners, senior executives, coroner, and professional regulators. That it was mentioned by all managers indicated its level of importance. This is captured by manager 1 who considered how a SII would be evaluated by the coroner:

If a report doesn't say what learning it's pulled out, that's when we'll give you a hard time or if we think you've completely missed the point. Manager:1

Related to this, carers' talk focussed upon how they felt that managers prioritised organisational reputation within the SII. Carers sensed learning was generated from SIIs, but were concerned about the implementation:

They're [managers] pushing the problem away and then refusing to let the psychiatrist and the team leader of the community team come to the inquest (…) it's really clear from the investigation that they have a role to play in what went wrong, but they're not willing to stand up and answer questions. Carer:3

A clinician similarly aired suspicion that organisational reputation was being inappropriately prioritised:

It had been several weeks since I had done the assessment and somebody else had done [another assessment] since, but they weren't asked for a report (…) I think that it was because the contact that this person had with the patient wasn't documented very well (…) I was suspicious that they didn't want that being made too public so they were relying on my information. Clinician:3

#### 3.3.2 Clinical Governance Accountability

Managers talked of clinical governance processes they oversaw:

The patient safety review panel (…), that's where we track progress [and] monitor it, but then we might say ‘right well actually that's the second one I've seen, and I'm concerned because I'm seeing the same themes’. Manager:1

However, a carer questioned whether senior managers had met expected governance standards:

The other thing I'd just like to pick up on is, they have actually sort of chosen to put a lot of the blame on the agency's care coordinator, [but] there's lots of systemic problems in the Trust, lots of lack of systems and oversight from senior people. Carer:3

Given the various potential sources of blame faced by managers, we understood that their talk provided mitigation against this. It is perhaps understandable that managers' talk predominantly attributed the barriers to organisational learning to the quality of the investigators' approach. Yet their talk highlighted the sensitive nature of conversations that would be undertaken with clinicians as part of SIIs. They appeared uncertain how to attend to this.

#### 3.3.3 Investigator Approach

Manager 4's talk describes the approach taken by the investigating team in failing to acknowledge the complexity underpinning the death:

What you tend to find is the person is wrapped in a bubble and parked off over there because ‘it's too hard, it's too emotional, it's disrespectful’, it's ‘we can't challenge [a] clinical decision, it's inevitable’ (…). What we focus on is process, so we avoid the complexity. Manager:4

Several managers also spoke about the sensitivities for clinicians in examining their actions as having the potential for contributing to a death:

In the 20 years I've been (…) working around SI's it's always the same thing, people worried they're going to get struck off, they worry their going to end up in court (…), but do we need to build in that kind of review quite early, so if the biggest thing on your mind when an SI happens is ‘am I going to get into trouble?’, if in week one we can see clearly there isn't action to be taken against anybody (…) so communicating as we go, we're confident that nobody's acted wilfully, nobody's done anything wrong here (…) maybe that helps people? I don't know. Manager:2

Another manager's talk describes their concerns that clinicians' understandable ways of coping with the death may prevent opportunities for learning:

Sometimes there's a sense of inevitability about suicide, ‘there's nothing we could have done to prevent it’. There's (…) probably a very protective narrative [for] the clinical staff who are involved because you turn that on its head and open it up to ‘what could we have done to prevent this?’ You're accepting culpability in some way and that makes it feel very personal, and I can understand that for resilience, people won't be consciously thinking like this, but for your own resilience you need that barrier. Manager:4

#### 3.3.4 Avoidance of Critical Reflection on Professional and Organisational Responsibility

None of the managers who voiced concerns about the investigator's approach shared their plans of how the organisation might overcome clinician concerns. Managers' talk did not link this significant barrier in the process to the concerns they had shared about the limited learning generated by investigators. Therefore, it was the sensitivity of the conversations within SII's rather than investigators' approach per se which appeared as a key barrier to high‐quality organisational learning.

### 3.4 Investigators

Investigators hold high levels of accountability, and those we spoke to took ownership of the role but did not share fear of blame. However, managers from the host Trust did talk about their investigators appearing to experience fear of blame in relation to their role.

#### 3.4.1 Legal Accountability

Participating investigators' talk suggested they were sensitive to the needs of all those involved. They did not share any overt concerns about the potential for professional or organisational blame. They did nevertheless speak with some trepidation regarding attendance at coroner's court, but this was when a family held contrary views to the SII outcome. This was evident, but underplayed in the following:

It really depends on how you feel that it's gone with the family as to whether you're looking (…) forward to it or not. Investigator:3

There was contrast between investigator and manager accounts. This might be explained by them being from different organisations. Managers described their own investigators as having a range of anxieties around openness and transparency. The following manager shared a concern that their investigator had appeared fearful of blame from the coroner:

It's not down to them whether the reports says good things or bad things, they're purely investigators (…) they were holding these investigation reports close to their chest and being nervous about perhaps disclosing things that they felt perhaps cast the Trust in a poor light. Manager:1

#### 3.4.2 Professional Accountability

One manager spoke empathically of investigators being fearful of blame when their work was being critiqued:

When there's research or scrutiny of our investigation processes, it's their work that is looked at and I don't think they're immune to that fear of blame, it's natural. Manager:5

Another manager spoke of investigators appearing anxious that they would be held responsible for front‐line clinician's actions:

This individual struggled particularly (a) with admitting we'd done something wrong as an organisation which is interesting and (b) that it would be perceived that it was her (…) but somebody else's actions were (…) not to the standard we would expect [of] our organisation. Manager:1

Some managers (above) appeared perplexed and appeared to have a lack of empathy, stating that their role was purely to focus on what could be learned.

Our reading made sense of investigators' talk as serving to mitigate against the fear of blame arising in relation to SIIs. We use this to understand why investigators talk conveyed a quiet confidence in their investigative practice, and that the organisation was not culpable.

#### 3.4.3 Avoidance of Critical Reflection on Investigative Effectiveness

Investigators' talk contrasted with accounts from managers and clinicians which had raised concerns about the quality of learning. They did not offer reflection on the quality of learning generated by their approach. This was despite all investigators agreeing with the following comment about not finding a root cause in the SII:

I've not yet had a root cause, I don't know if anybody else has? Investigator:1

When they spoke of identifying contributory factors rather than a root cause, they also acknowledged these were not seen as likely to have been preventative:

Often the learning lessons relate to things that could be improved but not in the sense that that would have prevented the suicide. Investigator:1

In further contrast to managers' accounts, investigators' talk minimised clinician anxieties as a potential barrier to openness within SIIs:

People [clinicians] find themselves in new situations, and that creates anxiety. But I've never (…) felt that there's a barrier, as in they're not telling me things. Investigator:1

#### 3.4.4 Avoidance of Professional Curiosity

Investigators talk about why patients may have decided to take their own lives did not include the psycho‐social context of patients' lives, or the quality of the therapeutic relationship. System‐level factors were deprivileged in favour of patient unpredictability and patient self‐determination. Instead, investigators explained that they were looking for evidence of suicide risk from patients' disclosures to clinicians. Below, an investigator recalls a suicide some days after an in‐patient discharge, evoking a certainty that suicide risk was *not* apparent:

There was no evidence of self‐harm (…) He said that he was fine (…) he'd (…) reflected on his life and just coming in [as an in‐patient], it being a bit of a wake‐up call (…) he talked about things he was going to do looking forward. Investigator:1

There was also a strong notion of inevitability regarding the nature of suicide in investigators' accounts:

I think regardless of whether we had taken his medication off him or not, his frame of mind, from what he was saying to us, he would have changed it to a different method; he would have killed himself. Investigator:2

Within accounts exploring risk assessment, none of the investigators considered raising the approach taken to risk assessment itself as a contributory factor:

People have done very good risk assessments and you can see that the judgements have been informed by information that they've gathered from everywhere and it's still wrong because they've gone and done it [taken their life] (…) you have to conclude that their assessment that the risk was low was reasonable based on what they knew, but this person still killed themselves. Investigator:1

When describing their approach to exploring the likely contributing factors to the death, investigators defaulted to (staff) adherence to local policy, procedures and national clinical guidelines:

It's not just the risk assessment, it's the care planning stuff, the staying safe [safety plans], the comprehensive assessment, the MDT's [multi‐disciplinary team's], some of the teams operate traffic light systems as well in terms of risk. Investigator:1

## 4 Discussion

Learning from suicides is complex and uncertain. In our discussion, we attend to a need for an improved focus upon learning and accountability rather than apportioning blame. Our findings, which cut across all participant groups, are novel and important and should act as a rallying call to attend to the fear of blame within ACMHS SIIs. Managers and investigators were not only protecting themselves but the organisation. Clinicians, and to a lesser extent carers, appeared protective of themselves in the understandings being developed within SIIs by their avoidance of critical reflection. We made sense of their talk as understandable safety‐seeking (conscious or unconscious) with their circumstances. Findings indicate that mitigation against fear of blame, which relates to differing roles and responsibilities, can open up or close down the ways in which the SIIs can be experienced. Consequently, the patient safety agenda and the key plank of suicide prevention must address fear of blame in order to enhance the quality of organisational learning to inform service‐level improvement.

Fear of blame is recognised as long standing issue in health services (Department of Health 2000). Our findings support those reported by HSSIB (2025) when exploring learning processes in relation to inpatient and post discharge deaths. Their report identifies the continued presence of a blame culture as a barrier to organisational learning. Indeed, one of their participants state: ‘healthcare professionals working in mental health are trained to hold anxiety, however when very tragic deaths occur there is an observable blame shifting and of pushing responsibility in every other direction but ourselves, whilst holding a toxic positivity that everything is OK around here’ (HSSIB 2025: s2.1.41). This quote captures the pushing away of blame on to others that we understand to be present in our participants' talk. Our findings also support the observations made by Cohen (2013) which draws upon a number of theories to explain the anxiety management processes within mental health staff in response to SIIs. Cohen (2013) not only highlights the cultural and organisational processes underpinning SIIs which may be influencing staff responses, but also feelings of self‐blame that can arise in response to suicide itself. Gibbons (2025) has explored self‐blame within carers and clinicians' post‐suicide and claims that it is a normal aspect of grieving. Consequently, there needs to be acceptance that the full eradication of self‐blame will not be possible.

Clinicians trod a vulnerable line between their awareness of the realities of clinical practice and the need to respond to the demands of SIIs, and it is unsurprising that they have been identified as second victims (Holden and Card 2019) in such tragic events. Clinicians' tendency to advocate self‐management is discussed by Brown (2021) as ‘responsibilising’. We argue that this response demonstrates emotional dysregulation specific to the enduring stress of managing suicidality, as described by Smith et al. (2015), and therefore demands that SIIs are sensitive and supportive. Furthermore, we claim that dysregulation is compounded by moral injury (Jameton 2017) that clinicians face in their roles working within pressured services. However, clinicians did not talk about their feelings of self‐blame that are reported by Gibbons (2025). Our understanding of clinicians' talk is that it may be related to a need to create a sense of personal and professional safety within the SII.

Carers accounts evidenced instances of epistemic injustice during the care of their loved ones (Okoroji et al. 2023). Notwithstanding their integrity as those closest to the victim, we were drawn to the apparent certainty held within carers' words conveying that if they had been listened to, the death could have been preventable. Gibbons (2025) also reports carers experiencing self‐blame as a response to suicide, but this was not mentioned by carers we spoke to. Gibbons (2025) discusses how carers seeking to avoid self‐blame could be likely to re‐direct this towards mental health services, which may have been the case for carers in our study. Interpersonal difficulties, including partners and family members, have been identified as pertinent for understanding suicidality (Chu et al. 2017). It is therefore important to consider how carers talk; mitigating a fear of blame represents a significant barrier for organisational learning.

We propose Managers' talk indicated a subtle sense of protectionism, mindful of their organisation incurring reputational damage. These findings concur with those reported by Ramsey et al. (2025) and Gibbons (2025). They attributed the problems identified in the quality of SIIs stemming from investigators' methods rather than anything associated with the wider organisational culture. Again, limiting organisational learning. They recognised clinician anxiety was a barrier within learning processes but did not associate this with the limited learning they described being generated by investigators. However, the experience of managers within SIIs under ACMHS's represents a gap in the literature.

Investigators strictly followed the SII process which could suggest a protective mechanism, in part safeguarding their own role, but also those of clinicians and the organisation. This reflects Cohen's (2013) position about objectivity being prioritised over subjectivity in the SII. Smith et al. (2015) discuss the reliance upon predictive and checklist approaches to suicide RA as a system anxiety management strategy. Despite recommendations to deprivilege predictive approaches (National Collaborating Centre for Mental Health 2018; National Confidential Inquiry into Suicide and Safety in Mental Health 2018; National Institute for Health and Care Excellence 2022) investigators did not appear to undertake a critical appraisal of the dominant approach taken to risk assessment, the unique issue of patient unpredictability in suicide, instead defaulting to self‐determination as an investigatory backstop. In a study which included mental health service investigators, Ramsey et al. (2025:10) highlight the complex nature of their roles with inadequate support whilst ‘shouldering unmanageable responsibility’. The investigators in our study did not report any fear of blame. However, we have made sense of this as arising from their use of mitigation to defend against any issues regarding the quality of the SII, and the organisation as being accountable for the suicide and therefore limiting opportunities for high‐quality learning.

A cogent strategy is needed to attend to the various mitigations which we understand were a response to the fear of blame across all participant groups in order to achieve high quality organisational learning. Turner et al. (2020, 2022) have demonstrated evidence of the impact of implementing a RJC, underpinned by a ZSF as part of an open culture, and is inclusive of carers. Their approach, however, does not identify the needs of managers or investigators as a specific group warranting attention. The PSIRF (NHS England 2022) suggests inroads into a restorative position, although it is not mental health specific and requires evaluation. The broader systems surrounding the deceased, including mental health services and beyond, should not be ignored. Indeed, Hawton et al. (2022) highlight that system anxiety can be exacerbated by the additional scrutiny from regulatory bodies, including coroners' inquiries. This has been discussed by Cohen (2017) and we are in agreement that there needs to be a whole systems approach to allow the improvement of the quality of learning generated from SII's.

## 5 Strengths and Limitations

This research took place as England's NHS was transitioning at scale to PSIRF (NHS England 2022), which may have influenced some participants' views.

It is possible participants experienced difficulties regarding open disclosure in this study. However, our readings did not indicate this. We acknowledge that the sensitive nature of the topic appeared to affect recruitment. We addressed this by recruiting across organisations which prevented an in‐depth study of one organisational culture. This should not, however, detract from the findings which form novel and valuable additions to a poorly researched but essential aspect of patient safety in ACMHS. We acknowledge the small size of this study whilst highlighting the synergistic impact of being able to cut across the four participant groups together.

## 6 Conclusion

This small exploratory study offers a fresh insight into the views of how key people, including carers, in SIIs are influenced by a fear of blame that influences their experience of SIIs. We argue this has implications for further research into the quality of organisational learning generated to inform future suicide prevention. Addressing fear of blame and its mitigation indicates a need for process change but also culture change, which inevitably demands effective leadership. To be clear of our position, we concur that investigation processes should not be seeking to explore who is to blame, but simply to make sense of what may have contributed to the suicide.

## 7 Relevance for Clinical Practice

Suicide investigation processes may wish to consider how fear of blame and mitigations limit the quality of organisational learning. Given the existing patient safety agenda, we advocate an inclusive approach to reach all those involved in SII processes, including carers. Continued professional development is well‐suited to this task and could bring carers and all staff members together in order to identify possible solutions. An inclusive, facilitatory leadership style would also be essential to create the necessary organisational culture for effective organisational learning. Given the exploratory nature of this study, further qualitative research is required to develop the conceptual foundations of the fear of blame and mitigation as discussed here.

## Author Contributions

All authors contributed to the formulation of the research question and research design. With oversight from author G.A., authors H.H. and T.S. undertook the research, analysed the data and wrote the final draft. The review was critically reviewed and approved by all authors.

## Conflicts of Interest

The authors declare no conflicts of interest.

## Supporting information


**Data S1:** inm70136‐sup‐0001‐Supinfo1.docx.

**Data S2:** inm70136‐sup‐0002‐Supinfo2.docx.

**Data S3:** inm70136‐sup‐0003‐Supinfo3.docx.

**Data S4:** inm70136‐sup‐0004‐Supinfo4.docx.

**Data S5:** inm70136‐sup‐0005‐Supinfo5.docx.

## Data Availability

Data not available due to participant consent. Participants of this study did not give written consent for their data to be shared publicly, so due to the sensitive nature of the research supporting data is not available.
