# Supplementary material for: Suicide Investigations in Adult Community Mental Health Services: Mitigation of the Fear of Blame as a Barrier to Organisational Learning
Source: Int J Ment Health Nurs. 2025 Sep 4;34(5):e70136. doi: 10.1111/inm.70136 (PMC12409766; doi:10.1111/inm.70136)
Supplement: Supplementary file 1 — Data S1: inm70136‐sup‐0001‐Supinfo1.docx. [file INM-34-0-s002.docx]

**Consolidated criteria for reporting qualitative studies (COREQ): 32-item checklist (adapted from Tong et al (2007)).**

| **No** | **Item** |  |  | **Page/section** |
| --- | --- | --- | --- | --- |
|  | Domain 1: Research team and reflexivity |  |  |  |
|  | Personal Characteristics |  |  |  |
| 1 | Interviewer/facilitator | Which author/s conducted the interview or focus group? | HH and TS conducted the interviews and focus groups. | Page 4 Data collection |
| 2 | Credential | What were the researcher’s credentials? E.g. PhD, MD | The researchers’ credentials are as follows: HH: DClinPsychol  TS: PhD  GA: PhD | Submission title page |
| 3 | Occupation | What was their occupation at the time of the study? | HH: Service Evaluation Lead TS: Assistant Professor  GA: Emeritus Professor | Submission title page |
| 4 | Gender | Was the researcher male or female? | The researcher’s genders are as follows:  HH: female  TS: male  GA: male | n/a |
| 5 | Experience and training | What experience or training did the researcher have? | All authors have previous experience of qualitative research to at least Doctoral level. HH and TS are experienced mental health professionals. GA is an experienced physical health nurse. | n/a |
|  | Relationship with participants |  |  |  |
| 6 | Relationship established | Was a relationship established prior to study commencement? | Previous relationships with the authors are undisclosed to safeguard anonymity. | n/a |
| 7 | Participant knowledge of the interviewer | What did the participants know about the researcher? e.g. personal goals, reasons for doing the  research | Previous relationships with the authors are undisclosed to safeguard anonymity. All participants were made aware of who the researchers were within the informed consent process. | Page 4 Data collection |
| 8 | Interviewer characteristics | What characteristics were reported about the interviewer/facilitator? e.g. Bias, assumptions, reasons and interests in the research topic | The characteristics of each author have been reported. | Submission title page |
|  | Domain 2: study design |  |  |  |
|  | Theoretical framework |  |  |  |
| 9 | Methodological orientation and theory | What methodological orientation was stated to underpin the study? e.g. grounded theory,  discourse analysis, ethnography, phenomenology, content analysis | The study was a multi-method qualitative study. A thematic analysis was undertaken on the data. | Design Page 3.  Data analysis Page 5 |
|  | Participant selection |  |  |  |
| 10 | Sampling | How were participants selected? e.g. purposive, convenience, consecutive, snowball | Purposive sampling was used. | Design Page 3 |
| 11 | Method of approach | How were participants approached? e.g. face-to-face, telephone, mail, email | Clinicians: invited by email.  Investigators: invited by their managers.  Carers: invited by letters, local and national support groups.  Senior managers: invited by email. | Design Page 3 and 4 |
| 12 | Sample size | How many participants were in the study? | Carers: 6  Investigators: 3  Senior Managers: 6  Clinicians: 4 | Design Page 3 and 4 |
| 13 | Non-participation | How many people refused to participate or dropped out? Reasons? | From an expression of interest;  Investigators: One investigator was unable to attend the focus group due to sickness.  Clinicians: One clinician was unable to attend the focus group due to a personal emergency.  Carers: None dropped out or refused.  Senior managers: None dropped out or refused. | n/a |
|  | Setting |  |  |  |
| 14 | Setting of data collection | Where was the data collected? e.g. home, clinic, workplace | Data was collected via online meetings. | Data collection Page 4 |
| 15 | Presence of non-participants | Was anyone else present besides the participants and researchers? | Our carer representative KD was present for our carer focus group. | Data collection Page 4 |
| 16 | Description of sample | What are the important characteristics of the sample? e.g. demographic data, date | All of our participants had to have experience of investigation processes within the last two years. | Design Page 3 |
|  | Data Collection |  |  |  |
| 17 | Interview guide | Were questions, prompts, guides provided by the authors? Was it pilot tested? | Participant specific topic guides were prepared by the researchers. KD reviewed the carers topic guide and feedback was included in the final version. | Data collection Page 4 |
| 18 | Repeat interviews | Were repeat interviews carried out? If yes, how many? | No | n/a |
| 19 | Audio/visual recording | Did the research use audio or visual recording to collect the data? | Interviews and focus groups were video recorded. | Data collection Page 4 |
| 20 | Field notes | Were field notes made during and/or after the interview or focus group? | No | n/a |
| 21 | Duration | What was the duration of the interviews or focus group? | Focus groups and interviews were between 60-90 minutes. | Data collection Page 4 |
| 22 | Data saturation | Was data saturation discussed? | Data saturation was not relevant to our design. | n/a |
| 23 | Transcripts returned | Were transcripts returned to participants for comment and/or correction? | No | n/a |
|  | Domain 3: analysis and findings |  |  |  |
|  | Data Analysis |  |  |  |
| 24 | Number of data coders | How many data coders coded the data? | HH and TS undertook the thematic coding. GA adjudicated any disagreements. | Data analysis Page 5 |
| 25 | Description of the coding tree | Did authors provide a description of the coding tree? | HH and TS used the process described in table 1 to arrive at the coding tree. | Data analysis Page 5  Results Page 6 |
| 26 | Derivation of themes | Were themes identified in advance or derived from the data? | Themes were derived from the data. | Data analysis Page 5 |
| 27 | Software | What software, if applicable, was used to manage the data? | No | n/a |
| 28 | Participant checking | Did participants provide feedback on the findings? | No | n/a |
|  | Reporting |  |  |  |
| 29 | Quotations presented | Were participant quotations presented to illustrate the themes / findings? Was each quotation identified? e.g. participant number | Yes | Results Pages 5-14 |
| 30 | Data and findings consistent | Was there consistency between the data presented and the findings? | Yes there was consistency between the data and the findings. | Results and Discussion Pages 5 – 18 |
| 31 | Clarity of major themes | Were major themes clearly presented in the findings? | Yes major themes were clearly articulated in the findings. | Results Pages 5-14 |
| 32 | Clarity of minor themes | Is there a description of diverse cases or discussion of minor themes? | No there was no discussion of the diverse cases or discussion of minor themes | n/a |
