# Supplementary material for: Suicide Investigations in Adult Community Mental Health Services: Mitigation of the Fear of Blame as a Barrier to Organisational Learning
Source: Int J Ment Health Nurs. 2025 Sep 4;34(5):e70136. doi: 10.1111/inm.70136 (PMC12409766; doi:10.1111/inm.70136)
Supplement: Supplementary file 2 — Data S2: inm70136‐sup‐0002‐Supinfo2.docx. [file INM-34-0-s005.docx]

**Focus group topic guide: Investigators**

Introduction

- Thank for joining the group and offering to take part in the study.
- Group facilitators to introduce themselves and invite group members to introduce themselves.
- Recap information sheet and ground rules for the focus group to confirm they are still comfortable with taking part.
- Acknowledge potential for emotional impact and they can ask at any point to take a break which can be supported by a group facilitator if they wish.
- Explain they are free to ask questions at any stage during the focus group.

Topis/questions

1. Opening question/context

- Could you describe your involvement in the SII process in relation to suicides that occur within the Trust?

1. Explore the use of theoretical or conceptual models/published standards in the investigation process

- Do you have an awareness of what informs the current approach to investigation process?
- Is there an evidence base?
- Is there a framework or model of any kind?

1. Examine the various factors (for example, contributory and bio-psycho-social) which are considered in the SII regarding suicide risk and how these inform organizational learning.

- What factors do you consider when exploring how suicide risk was approached by the service?

1. Identify the barriers and facilitators to an effective investigation of care provision

- How are staff members, family members/carers involved in the process?
- How is work as done and work as imagined grappled with?
- Do you feel that the investigations capture the complexities of the situation (re the service and re risk assessment)?
- Do you view them as effective in generating the organizational learning that is essential to the service in relation to service factors as well as in relation to risk assessment?
- What gets in the way do you think? What helps?
- Do you perceive the SII process to be open and transparent? In relation to how carers are involved? Do you experience MH clinicians being open in the process? And does the process feel transparent at the stage of involvement with senior managers and what is shared with the coroners?

1. Are there any aspects of the process that we haven’t covered that you would like to mention?
2. End of interview – thank you. Offer follow up conversation if they feel they have any support needs.
