# Supplementary material for: Suicide Investigations in Adult Community Mental Health Services: Mitigation of the Fear of Blame as a Barrier to Organisational Learning
Source: Int J Ment Health Nurs. 2025 Sep 4;34(5):e70136. doi: 10.1111/inm.70136 (PMC12409766; doi:10.1111/inm.70136)
Supplement: Supplementary file 3 — Data S3: inm70136‐sup‐0003‐Supinfo3.docx. [file INM-34-0-s001.docx]

**Focus group topic guide: Carers**

Introduction

- Thank you for joining us today and offering to take part in this study.
- Group facilitators to introduce themselves
- Recap information sheet and ground rules for the focus group to confirm they are still comfortable with taking part.
- Acknowledge potential for emotional impact and they can ask at any point to take a break which can be supported by a group facilitator if they wish.
- Feel free to ask questions at any stage during the focus group.
- Invite group members to introduce themselves to the group.

Topic/questions

1. Opening question.

- Would anyone like to share something about how they felt about taking part in this study?

1. Carers views on SII’s in relation to suicide.

- Tell me about your experience of the investigation.
- Was there anything you felt the investigation didn’t include that you felt was important?
- Were any questions that you had answered?
- Did you feel that your views of the care that had been provided were incorporated into the investigation?
- Did you see anything that you felt blocked/got in the way of the investigation in understanding what had happened?
- Did the investigation feel transparent and open?

1. Carer’s views upon how the investigation explored suicide risk.

- How did the investigation look at how the service assessed if your relative was at risk of deciding to end their life? Do you have a sense of specific areas (e.g. anything the person may have said or did, how they were responding to treatment) that were considered? Was there anything you felt that they did not take into account?

1. Carers views upon views of investigations as a tool to generate organisational learning.

- What did you make of the action/learning points that were generated?
- Were the (action points) what you expected? Did they fit your understanding that you may have had around the things that you felt had led your relative to ending their lives?
- Were you left feeling confident that what was learnt by the investigation would help to prevent the death of other users of our services?

1. Are there any aspects of the process that we haven’t covered that you would like to mention?
2. End of interview – thank you.

- Reminder that we will be contacting all group members to seek to ensure their wellbeing within the next 24-72 hours.
