# Supplementary material for: Suicide Investigations in Adult Community Mental Health Services: Mitigation of the Fear of Blame as a Barrier to Organisational Learning
Source: Int J Ment Health Nurs. 2025 Sep 4;34(5):e70136. doi: 10.1111/inm.70136 (PMC12409766; doi:10.1111/inm.70136)
Supplement: Supplementary file 4 — Data S4: inm70136‐sup‐0004‐Supinfo4.docx. [file INM-34-0-s004.docx]

**Focus group topic guide: Clinicians**

Introduction

- Thank for joining us the group and offering to take part in this study.
- Group facilitators to introduce themselves and invite group members to introduce themselves.
- Recap information sheet and ground rules for the focus group to confirm they are still comfortable with taking part.
- Acknowledge potential for emotional impact and they can ask at any point to take a break which can be supported by a group facilitator if they wish.
- Explain that they are free to ask questions at any stage during the focus group.

Topic/questions

1. Opening question/context

- Would anyone like to share something about how they felt about taking part in this study?

1. Clinician views on SII’s in relation to suicide.

- What were your thoughts about the investigation?
- Was there anything you felt the investigation didn’t include that you felt was important? Were any questions that you had answered?
- Did you feel that the investigation reflected the complexities of the situation?
- Did you see anything that you felt helped or blocked/got in the way of the investigation in understanding what had happened?
- Did the investigation feel transparent and open? Did you feel safe in being open about your own practice?

1. Clinician views upon how the investigation explored suicide risk.

- How did the investigation explore the approach taken to the assessment of suicide risk? Do you have a sense of specific areas they were looking at? Was there anything you felt that was not taken into account? Anything that was given less attention?

1. Clinician views of investigations as a tool to generate organisational learning.

- What did you make of action points that were generated?
- Did you agree with the conclusions that were made? Did they fit your understanding that you may have had around the things that you felt had led to the service user ending their lives?
- Were you left feeling confident that what was learnt by the investigation would help to prevent the death of other users of our services?

1. Are there any aspects of the process that we haven’t covered that you would like to mention?
2. End of interview – thank you.

- Reminder that we will be contacting all group members to seek to ensure their wellbeing within the next 24-72 hours.
