# Supplementary material for: Suicide Investigations in Adult Community Mental Health Services: Mitigation of the Fear of Blame as a Barrier to Organisational Learning
Source: Int J Ment Health Nurs. 2025 Sep 4;34(5):e70136. doi: 10.1111/inm.70136 (PMC12409766; doi:10.1111/inm.70136)
Supplement: Supplementary file 5 — Data S5: inm70136‐sup‐0005‐Supinfo5.docx. [file INM-34-0-s003.docx]

**Interview topic guide: Senior Manager**

I

ntroduction

- Provide thanks for joining the meeting and offering to take part in the study.
- Recap information sheet to confirm they are still comfortable with taking part.
- Acknowledge potential for emotional impact and they can ask at any point to take a break which can be supported by a group facilitator if they wish.
- Explain that they are free to ask questions at any stage during the interview.

Topics/questions

1. Opening question/context

- Could you describe your involvement in the SII process in relation to suicides that occur within the Trust?

1. Explore the use of theoretical or conceptual models/published standards in the investigation process

- Do you have an awareness of what informs the current approach to investigation process?
- Is there an evidence base?
- Is there a framework or model of any kind?

1. Examine the various factors (for example, contributory and bio-psycho-social) which are considered in the SII regarding suicide risk and how these inform organizational learning.

- How is the approach taken to *suicide risk* examined or evaluated?
- What factors do you see being considered as possible contributory factors within the service?

1. Identify the barriers and facilitators to an effective investigation of care provision.

- Do you view them as effective in generating the organizational learning that is essential to the service in relation to service factors as well as in relation to risk assessment?
- What gets in the way do you think? What helps?
- Do you perceive the SII process to be open and transparent? In relation to how carers and staff are involved? And does the process feel transparent at the stage of involvement with senior managers and what is shared with the coroners?
- Do you feel that the investigations capture the complexities of the situation (re the service and risk assessment)?
- How do you balance blame and accountability in the investigation process?

1. Are there any aspects of the process that we haven’t covered that you would like to mention?
2. End of interview – thank you. Offer follow up conversation if they feel they have any support needs.
